# Exploring the knowledge and practice of calcium channel blocker overdose management among South African Emergency Medicine doctors

**DOI:** 10.1016/j.afjem.2026.100970

**Published:** 2026-04-01

**Authors:** Nakita Pluymers, Victoria Stephen, Mike Wells

**Affiliations:** a Division of Emergency Medicine, Faculty of Health Sciences, School of Clinical Medicine, University of the Witwatersrand, Johannesburg, South Africa; b Department of Emergency Medicine, Far East Rand Hospital, Johannesburg, South Africa; c Department of Emergency Medicine, Charles E Schmidt College of Medicine, Florida Atlantic University, Boca Raton, USA

**Keywords:** Calcium channel blocker, Management, Emergency department, Knowledge, Practice, Overdose

## Abstract

**Introduction:**

Calcium channel blocker (CCB) agents have a high mortality rate in overdose (OD) in comparison to other cardiovascular agents. Literature on the emergency management of severe CCB OD is scarce and largely restricted to high-income countries. Within the African context, enhanced knowledge regarding CCB ODs has the potential to reduce mortality and promote adherence to clinical guidelines, provided that practice-related barriers are concurrently addressed. This study aimed to assess the knowledge and practice of emergency department (ED) doctors in managing a CCB OD, use of high dose euglycaemic insulin therapy (HIET), and barriers encountered in CCB OD management.

**Methods:**

We conducted a prospective cross-sectional mixed methods survey among 119 South African emergency medicine doctors, from June 2021 until October 2021, to assess their knowledge and practice of managing CCB OD’s, using a convenience sampling method. It was a self-administered electronic survey containing 39 questions, including demographics and a combination of open, closed and case-based multiple-choice questions. The closed and multiple-choice questions were analysed using descriptive statistics and chi-square tests to examine associations between demographic variables and responses. Open-ended questions were analysed using thematic content analysis.

**Results:**

Of the 119 doctors who participated in the survey, 59 were public ED doctors, 30 were registrars (specialist trainees in emergency medicine), and 30 worked in private EDs. The mean (SD) age of participants was 32.9 years (6.4). The mean (SD) score of all participants was 54% (13.3), which was the lowest for public ED doctors (48%) and significantly higher for private ED doctors (58.4%) and registrars (60.1%). Forty-eight percent of participants didn’t know of any guidelines to treat a CCB OD. When asked about their current practice in managing CCB OD, regarding their choice of vasopressor or inotrope, 97.5% (*n* = 116) of all participants chose adrenaline. For the total group, the most common barriers found when using HIET were the unavailability of infusion pumps (*n* = 72, 60.5%) followed by understaffing in the unit (*n* = 51, 42.9%) and the resuscitation area being full (*n* = 39, 32.8%).

**Discussion:**

This study has highlighted the need to address ED doctors' knowledge gaps on CCB OD management, further emphasising the importance of clinical toxicology education in South Africa. Although up-to-date guidance is freely accessible, its consistent use appears limited, emphasising the need for improved dissemination and integration into everyday emergency medicine and toxicology practice.

## African relevance


 

• Calcium Channel Blockers are common anti-hypertensive agents used in the African population with a high mortality rate from overdose.
• Literature on the emergency management of calcium channel blocker overdoses is limited to high-income countries, and there is a paucity of data on the African continent.
• While international protocols provide valuable guidance, their applicability may be limited by resource constraints in African settings, emphasising the need for context-appropriate approaches.
• Clinical toxicology education is pertinent in Africa and needs to be highlighted in Emergency Department teaching and training.

## Introduction

Calcium channel blockers (CCB) are regularly used antihypertensive agents. When used in overdose (OD), they can result in circulatory shock and multi-organ failure, which may be fatal [1]. They are responsible for more fatalities than any other cardiovascular agent in the USA, resulting in a 42% mortality rate in one such report [2]. Deliberate self-poisoning is also a significant public health issue in low- and middle-income countries [3,4]. A South African (SA) study found that cardiovascular medicines were the second most common ingestion [4]. CCBs are commonly used antihypertensive agents across Africa [5]; therefore, doctors on the continent ought to be proficient in managing CCB ODs.

The treatment of a severe CCB OD can be challenging for emergency doctors as often a multimodal therapeutic approach is needed [6]. Such an approach can be divided into supportive treatments, including airway, breathing, and circulation management; and specific treatments, including intravenous calcium, vasopressors, and high-dose insulin euglycaemic therapy (HIET). International and SA guidelines have encouraged the use of HIET in severe CCB ODs [1,7]. Variability in emergency clinician awareness of guideline-based treatment, including HIET, may lead to suboptimal outcomes [8]. An international expert consensus guideline was published to assist in the treatment of CCB ODs, thereby reducing discrepancies in physician practice [7]. Following such a guideline has also shown improvements in cardiac output, blood pressure, and possibly survival [7].

Despite international guidelines on CCB OD management being available, guidelines are not always followed in practice. This is known as the knowledge-practice gap [9]. For example, in Canada, only 42% of CCB poisonings are managed according to poison control advice, largely due to a lack of physician training in managing overdoses and limited resources [8]. The knowledge-practice gap must be narrowed, particularly in managing life-threatening emergencies. Addressing this issue may be achieved by assessing clinician knowledge and practice, which can be used to identify training needs, refine clinical protocols, and ultimately improve the management of CCB OD. Further narrowing this gap includes addressing practice barriers to managing this high acuity toxicological emergency. In resource-limited settings, the absence of systematic identification of practice barriers hampers the effective implementation of evidence-based interventions for CCB overdose. Recognizing and addressing these barriers is therefore critical to developing implementation strategies that promote physician adherence and ultimately ameliorate patient outcomes [10].

There is a paucity of research on the knowledge and practices of ED doctors pertaining to CCB OD management in low- and middle-income countries. The aim of this study was to assess emergency department (ED) doctors' current knowledge and practices in managing CCB OD, as well as their use of HIET and any related practice barriers. This was achieved using a structured survey with open- and closed-ended questions.

## Methods

### Study design

This was a prospective observational cross-sectional study employing a mixed methods approach.

### Participants and setting

The study employed a convenience sampling method to recruit ED doctors in SA consisting of three groups: Emergency Medicine (EM) registrars (trainee specialists/residents), ED doctors working in private emergency departments and ED doctors working in EDs in state hospitals. For this study, registrars referred to specialist trainees in EM, equivalent to residents or specialty trainees in other countries. Doctors from other departments, interns rotating through EDs, and incomplete surveys were excluded. (Appendix A) Sample size calculation was based on the 5% significance level, 80% power and medium effect sizes (Cohen’s *d* = 0.4), for three subgroups, using paired statistical tests, which recommended a sample size estimation of 30 participants in each subgroup.

### Data collection

The survey was developed by the authors and evaluated against current evidence-based CCB overdose guidelines. It was pilot tested with doctors from other departments before distribution.

This prospective cross-sectional mixed-methods study used quantitative and qualitative approaches to explore clinicians' knowledge and practice of CCB overdose management through a 39-question survey (Appendix B). Survey questions assessing HIET familiarity and application were informed by thematic concepts from Brassard et al.'s qualitative study [10]. Additionally, perceived barriers to clinician HIET use were assessed through closed-ended questions.

The electronic survey using SurveyMonkey® was distributed via email and social media advertising to doctors working in EDs across public and private hospitals nationwide.(Appendix C). In-person visits to Gauteng EDs were also conducted to invite medical officers and registrars to participate via a survey link. The survey was open from June 2021 until October 2021, with reminders sent to encourage participation. The survey, disseminated via social media platforms, resulted in broad, country-wide visibility; thus, the university affiliation of registrars was not captured due to anonymity. No personal or identifiable information was collected, and participation was voluntary and anonymous. Responses were stored on the password-protected SurveyMonkey® platform, accessible only to the PI.

After survey completion, participants could access an optional Continuous Professional Development (CPD)-accredited webinar on evidence-based CCB overdose management, developed by the PI using current literature and international guidelines. Survey anonymity was preserved since webinar access occurred post-survey without researcher knowledge of participants.

### Data analysis

The data was exported onto a spreadsheet (Microsoft Excel, Microsoft Office 365, Microsoft Corporation). SPSS® Statistics version 27 was used to analyse the data. Descriptive statistics using bivariate analysis were conducted using appropriate parametric or non-parametric statistical tests, depending on the distribution and variables of the sample population. Associations between categorical variables were assessed using the Chi-squared test. Closed-ended questions received 1 point for each correct answer, with open-ended questions scored using predetermined criteria (Appendix D). Total scores generated an overall knowledge score. A one-way ANOVA was used to assess group differences. Open-ended barrier questions were analysed qualitatively for common trends. A p-value < 0.05 was considered statistically significant.

### Ethical considerations

The study was approved by the Witwatersrand University Human Research Ethics Committee (M201053). Participant anonymity was maintained, and data were accessible only to researchers.

## Results

### Participant characteristics

A total of 246 survey responses were received, of which 119 were complete and met the inclusion criteria for analysis. Incomplete responses and those not progressing beyond demographics were excluded.

### Demographics

The cohort of doctors who participated in the survey consisted of 59 public Emergency Department (ED) doctors, 30 registrars, and 30 doctors working in private EDs. The mean (SD) age of participants was 32.9 years (6.4), and the average years of experience in emergency medicine was 4.7 years (3.7) (Table 1).

**Table 1:** Demographics and Clinical Experience of Study Participants.

	Total % (*n* = 119)	Private % (*n* = 30)	Public % (*n* = 59)	Registrars % (*n* = 30)	P value
Age					
Under 30 years	26.9% (32)	10% (3)	40.7% (24)	16.7% (5)	0.004
30–34 years	53.8% (64)	70% (21)	37.3% (22)	70% (21)	
35 years or older	19.3% (23)	20% (6)	22% (13)	13.3% (4)	
Years of experience in the Emergency Department					
1 year	18.5% (22)	16.7% (5)	27.1% (16)	3.3% (1)	0.001
2–3 years	26.9% (32)	23.3% (7)	35.6% (21)	13.3% (4)	
4–5 years	30.3% (36)	30% (9)	15.3% (9)	60% (18)	
6+ years	24.4% (29)	30% (9)	22% (13)	23.3% (7)	

Over the past year, 42% (*n* = 50) of the sample population had not treated a CCB OD; 22.7% (*n* = 27) had only treated one, and 35.3% (*n* = 42) had treated two or more. Of note, 60% (*n* = 18) of doctors working in the private sector had not treated a single CCB OD, while 56.7% (*n* = 17) of the registrar group had managed two or more CCB ODs within the past year, *p* = 0.003. Forty-five percent (*n* = 54) of the group had used high-dose insulin euglycaemic therapy (HIET) in CCB OD management before, with more registrars than public ED doctors and private ED doctors having experience with HIET: 83.3% (*n* = 25) versus 32.2% (*n* = 19) and 33.3% (*n* = 10) respectively, *p* < 0.001.

### Knowledge

**Fig. 1:**
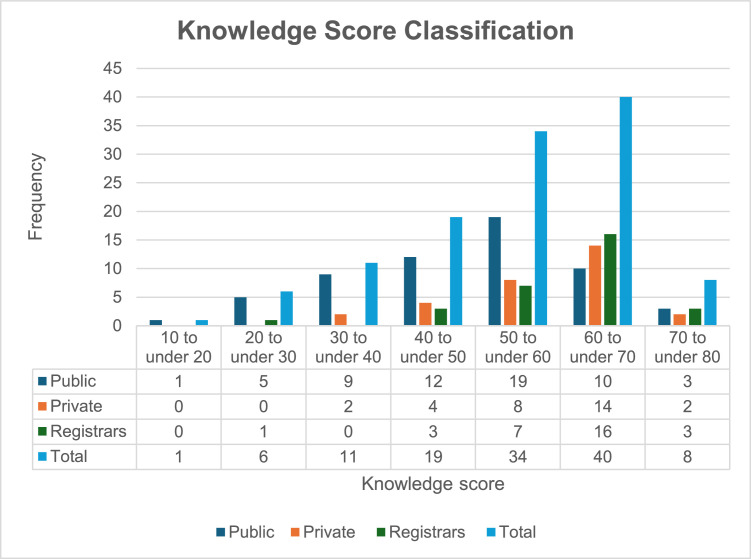
Frequency distribution of CCB overdose knowledge scores by participant groups.

The mean knowledge score of all participants was 54% (13.3) and the median was 55.9%. Overall, 33.6% (*n* = 40) of the group scored between 60 and 70 percent, see Fig. 1. The mean knowledge score was the lowest for public ED doctors (*n* = 59, 48%; median 51.5%) and significantly higher for private ED doctors (*n* = 30, 58.4%; median 61%) and registrars (*n* = 30, 60.1%; median 62.5%). There was a significant statistical difference in mean scores between registrar subgroup versus the public ED doctors; as well as private ED doctors versus the public ED doctors, *p* < 0.001 and *p* = 0.002. There was no statistical significance between knowledge score and different years of experience (*p* = 0.071).

When asked which guidelines they use to treat a CCB OD, 48% (*n* = 58) of the group didn’t know of any guidelines. Of those participants who did utilise guidelines, the most used were either international guidelines, the EMGuidance application or a combination of resources, see Table 2 below.

**Table 2:** Guidelines used to treat a calcium channel blocker overdose.

Total (*n* = 119)		Private % (*n* = 30)	Public % (*n* = 59)	Registrars % (*n* = 30)
Unsure/I don’t know	48.7% (58)	46.7% (14)	54.2% (32)	40% (12)
Afritox	0.8% (1)	3.3% (1)	0% (0)	0% (0)
International guidelines	10.1% (12)	13.3% (4)	5.1% (3)	16.7% (5)
EMGuidance	7.6% (9)	13.3% (4)	8.5% (5)	0% (0)
Hospital/Emergency Department guidelines	3.4% (4)	3.3% (1)	5.1% (3)	0% (0)
Life in the fast lane	4.2% (5)	3.3% (1)	5.1% (3)	3.3% (1)
National Department of Health guidelines	1.7% (2)	3.3% (1)	1.7% (1)	0% (0)
South African Medical Journal article, Stephen et al.	5% (6)	3.3% (1)	3.4% (2)	10% (3)
Other	5.9% (7)	3.3% (1)	10.2% (6)	0% (0)
>1 source	8.4% (10)	3.3% (1)	3.4% (2)	23.3% (7)
None	4.2% (5)	3.3% (1)	3.4% (2)	6.7% (2)

Most of the registrars (*n* = 29, 96.7%) responded that they learnt about HIET while working in the ED rather than from journal articles, textbooks, or medical school. A high percentage of public ED doctors (*n* = 13, 22%) only learnt about HIET while doing the survey. See Table 3.

**Table 3:** Where did ED doctors first learn about HIET.

Total (*n* = 119)		Private (*n* = 30)	Public (*n* = 59)	Registrars (*n* = 30)
Working in Emergency Department	68.9% (82)	56.7% (17)	61% (36)	96.7% (29)
Journal article	5% (6)	13.3% (4)	3.4% (2)	0% (0)
Colleagues	4.2% (5)	10% (3)	3.4% (2)	0% (0)
Medical school	2.5% (3)	0% (0)	5.1% (3)	0% (0)
Textbook	2.5% (3)	0% (0)	5.1% (3)	0% (0)
I haven’t heard about it before now	12.6% (16)	6.7% (3)	22% (13)	0% (0)
Other	3.4% (4)	10% (3)	0% (0)	3.3% (1)

### Practices

When asked about their current practice in managing CCB OD, regarding their choice of vasopressor or inotrope, 97.5% (*n* = 116) of ED doctors chose adrenaline. Dopamine (*n* = 1, 0.8%) and noradrenaline (*n* = 2, 1.7%) were less preferred by the group. When asked about whether they seek advice on the management of CCB ODs, 63.9% (*n* = 76) of participants stated they would consult an Emergency Medicine (EM) physician for advice in comparison to just 11.8% (*n* = 14) who would rather consult a poison centre, and 9.2% (*n* = 11) who would ask a more senior doctor on the floor, *p* = 0.03. Twenty percent (*n* = 6) of private ED doctors would seek advice from either an intensivist, an internal medicine consultant or a cardiologist.

### Barriers to high-dose insulin euglycaemic therapy

The barriers to initiating HIET in a CCB OD are described in Table 4. The most common barriers were the unavailability of infusion pumps (*n* = 72, 60.5%) followed by understaffing in the unit (*n* = 51, 42.9%), the resuscitation area being full (*n* = 39, 32.8%), the lack of an available consultant (*n* = 32, 26.9%), and the ED being busy (*n* = 29, 24.4%). There were no statistically significant differences between the subgroups.

**Table 4:** Barriers to initiating HIET in a calcium channel blocker overdose.

Factors	Total (*n* = 119)	Private (*n* = 30)	Public (*n* = 59)	Registrars (*n* = 30)	p value
General Barriers					
Unavailable Consultant	26.9% (32)	30% (9)	32.2% (19)	13.3% (4)	0.150
Lack of ICU beds	9.4% (35)	33.3% (10)	32.2% (19)	20% (6)	0.422
Unable to call the poison centre	5.9% (7)	6.7% (2)	8.5% (5)	0% (0)	0.269
CVP confidence	11.8% (14)	13.3% (4)	13.6% (8)	0% (0)	0.605
Busy ED	24.4% (29)	30% (9)	23.7% (14)	20% (6)	0.657
Full Resuscitation beds	32.8% (39)	30% (9)	32.2% (19)	36.7% (11)	0.852
Unavailability of infusion pumps	60.5% (72)	53.3% (16)	59.3% (35)	70% (21)	0.404
Understaffing in the unit	42.9% (51)	43.3% (3)	37.3% (22)	53.3% (16)	0.351

## Discussion

This first formal assessment of South African ED doctors' knowledge and practices in CCB overdose management revealed suboptimal knowledge scores with significant variability across clinician subcohorts. HIET awareness and application were particularly limited, highlighting persistent gaps between knowledge and clinical practice in toxicological emergencies and reinforcing the need for targeted education and guideline awareness.

The mean knowledge score was 54% which is slightly higher than an international study done by Monteith et al., with participants scoring 45.2% [11]. Monteith et al. also showed that Emergency Medicine (EM) trainees, our registrar equivalent, scored significantly better, achieving a score of 82.3% in comparison to CMOs (chief medical officers) of 65.4% [11]. This comparison between sectors was also observed in our study, demonstrating knowledge gaps and application difficulties, particularly in the public setting. Marks et al. highlighted that high mortality rates in acute poisonings are due to numerous factors, one being poor medical care, including the lack of toxicological expertise among health care providers [12]. Maude St-Onge emphasised that in managing unstable poisoned patients, doctors often report cognitive overload, and that better knowledge of cardiovascular drug toxicity would improve patient outcomes [13]. Doctors in public EDs were generally less experienced, which may have limited their exposure to structured academic training in toxicology. This could partly explain their lower knowledge scores, despite the presence of EM specialists across both sectors. Similarly, many private EDs employ junior doctors, including those immediately post-community service, and may not be run by EM specialists. In comparison, registrars train in emergency medicine for four years full-time in academic institutions; thus, they were expected to score above average on the knowledge component of the survey [14].

Although guidelines for the management of a CCB OD exist, our findings showed 48.7% of the group were not familiar with any guidelines, suggesting that awareness and consistent application of these guidelines remain limited in practice. Brassard et al. found that intensivists often face barriers when implementing HIET, even when evidence-based therapies are well established, including knowledge gaps, logistical challenges, or resource constraints [10]. This underscores a broader issue: the existence of effective guidelines does not guarantee their uptake in routine practice [15]. Therefore, the critical gap lies not in the absence of protocols but in the awareness, dissemination, and practical implementation of these recommendations in CCB toxicity, warranting further targeted research. The use of CPD-accredited educational interventions aligns with strategies described by Lang et al., who emphasise the value of continuing professional development in facilitating evidence uptake among clinicians [16]. This model also offers a foundation for future research to evaluate the impact of CPD-linked interventions on clinical behaviour and practice change.

In our study, 83.3% of registrars were more comfortable in starting HIET early when compared to public ED doctors (32.2%) and private ED doctors (33.3%). This could be due to their academic teaching, rotations through ICU’s or possibly more experience (56.7%) of having managed more than two CCB ODs over the past year. The selection of vasopressors used in a CCB overdose should be guided by the type of shock [13], thus both noradrenaline and adrenaline are recommended. Most participants opted to use adrenaline, which is readily available in all South African EDs, as it is freely available and included in the SA Essential Drugs List [17]. For the minority who opted for noradrenaline, it was either private doctors or registrars, as it is not widely available in the public sector.

A very low percentage of ED doctors would consult a poison control centre (PCC) when having to manage a CCB OD. According to the WHO, PCCs are a scarce resource in most African countries with only 17.2% of African countries having a PCC [12]. SA only has three Poisons Information Centres in the public sector, two of which are situated in the Western Cape [18]. Such resources are underutilised in SA and would assist as an additional resource of expert information in managing acute poisoning [21].

Beyond limited guideline familiarity, the practical barriers identified in our study impede the timely initiation of HIET, suggesting that bridging the knowledge gap also requires addressing systemic and logistical challenges. Among resources, participants most often lacked infusion pumps. Balaeni et al. found that only 53.9% of African clinicians always had access to syringe pumps versus 100% in high-income countries [19]. Understaffing further compounded these challenges, with few personnel available to monitor patients during the resource-intensive HIET. ED overcrowding and full resuscitation areas created additional practical obstacles, making it difficult to deliver complex care in a timely and safe manner. These findings align with Mazer-Amirshahi et al. and Kongchhep et al., demonstrating that resource limitations, staffing shortages, and high patient volumes impede specialised toxicology interventions and worsen patient outcomes [20,21] Overcoming these practical barriers is critical to ensuring that all patients with CCB OD receive timely, safe, and evidence-based care.

## Limitations

Due to the survey’s anonymous and widely distributed nature, the geographic representation of respondents couldn’t be determined. These findings provide valuable insight into current practice patterns; however, they may not be fully generalisable to all emergency clinicians in SA, given the potential for selection bias, and differences in practice settings. Caution should also be exercised in extrapolating the results to other countries or healthcare systems. While the survey underwent face and content validation by EM experts and pilot testing, it did not undergo formal validation. This may limit the ability to generalise the findings or assess internal reliability, although clinical relevance was ensured through expert review.

## Conclusion

This study has highlighted the need to address and bridge knowledge gaps on CCB OD management, emphasising the importance of clinical toxicology in SA and, in turn, underscoring the necessity for EM toxicology education. Although up-to-date guidance is freely accessible, its consistent use appears limited, emphasising the need for improved dissemination and integration into everyday EM and toxicology practice. Future research should meticulously examine barriers and facilitators to evidence-based care in the ED, assess how toxicology education can close the knowledge–practice gap, refine methodologies, and evaluate patient-centred outcomes of management strategies. Addressing these priorities will not only improve clinical practice and training but also patient safety in CCB OD emergency treatment.

## Dissemination of results

The results of this study were shared with the Emergency Medicine Division at the University of the Witwatersrand. The findings may be presented at local or international conferences in the future.

## CRediT authorship Contribution Statement

**NP:** Conceptualization, Methodology, Resources, Investigation, Formal analysis, Data curation, Writing -Original Draft, Visualization; **VS:** Conceptualization, Supervision, Writing – review & editing; **MW:** Project administration, Supervision, Validation, Writing – review & editing; **All authors** approved the version to be published and agreed to be accountable for all aspects of the work.

## Declaration of competing interest

The authors declare that they have no known competing financial interests or personal relationships that could have appeared to influence the work reported in this paper.

## Appendix Supplementary materials

Image, application 1

Image, application 2

Image, application 3

Image, application 4
