# Supplementary figures and images for: Exploring the knowledge and practice of calcium channel blocker overdose management among South African Emergency Medicine doctors
Source: Afr J Emerg Med. 2026 Apr 1;16(2):100970. doi: 10.1016/j.afjem.2026.100970 (PMC13087764; doi:10.1016/j.afjem.2026.100970)

## Appendix A: Cross-Functional Flow Chart

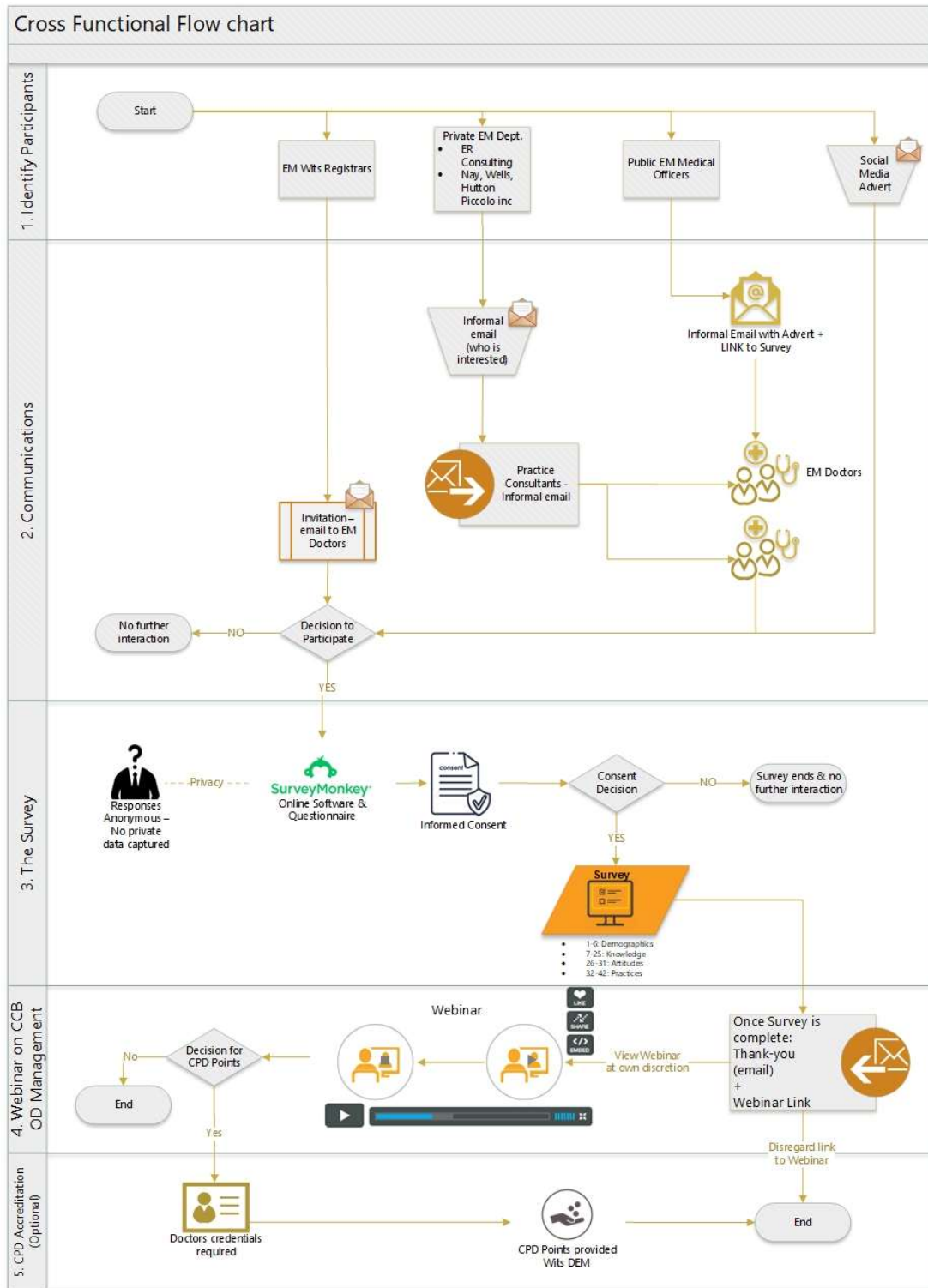

Supplement: Supplementary file 1 [file mmc1.pdf]
