# Supplementary material for: Exploring the knowledge and practice of calcium channel blocker overdose management among South African Emergency Medicine doctors
Source: Afr J Emerg Med. 2026 Apr 1;16(2):100970. doi: 10.1016/j.afjem.2026.100970 (PMC13087764; doi:10.1016/j.afjem.2026.100970)
Supplement: Supplementary file 2 [file mmc2.pdf]

Appendix B: Questionnaire with answers

Demographics

1. **Do you work as an Emergency Department (ED) doctor?**
   - ○ Yes: carry on with rest of survey
   - ○ No: please stop survey

2. **What is your age? (years)**

   Insert number

3. **Which ED do you work in?**
   - ○ Public
   - ○ Private

4. **Are you a:**
   - ○ Community service officer
   - ○ Medical officer
   - ○ Registrar
   - ○ Other: please specify.............................

5. **How many years have you worked in an Emergency department?**

   Insert number

Knowledge

Answers are highlighted

6. **With which of the following signs does a patient with toxic calcium Channel Blocker overdose clinically present with? Tick all that apply**
   - ☐ Hypertension
   - ☐ Hyperglycaemia
   - ☐ Hypotension
   - ☐ Miosis
   - ☐ Hyperthermia
   - ☐ Depressed level of consciousness
   - ☐ Hyponatraemia
   - ☐ Other: please state

7. **What is the minimum toxic dose of Amlodipine in a 70 kg adult?**
   - ○ 20 mg
   - ○ 0,1 mg/kg

- 10 mg
- 5 mg
- I don’t know

The following questions (Q8-11) relate to the following scenario:

8. **A 20-year-old female presents to the ED 12 hours after ingesting 60 tablets that were prescribed for her grandmother’s hypertension. The patient’s vitals are BP= 85/40 mm Hg, HR= 45 BPM, SPO2= 85% on RA and a GCS of 13/15.**

Which initial general measures would you start on this patient? Tick 2:

- ☐ Give activated charcoal
- ☐ Reliable intravenous access
- ☐ Gastric lavage
- ☐ Oxygenation using a non-rebreather mask
- ☐ Start an N-acetylcysteine infusion
- ☐ Cardiology consult

9. **After acquiring further history from the patient’s escort, they show you 2 empty 10mg Amlodipine boxes. The patient repeat vitals are: BP= 84/42 mm Hg, HR=38 BPM, SPO2= 88% on a non-rebreather mask and her GCS has dropped to 7/15. An arterial blood gas was done and shows the following results: pH: 7.12, PaCO2: 58mmHg, PaO2: 43mmHg, Na: 140 mmol/l, K: 4.2 mmol/l, Cl: 101 mmol/l, Ca: 0.89 mmol/l, glucose: 16 mmol/l, lactate: 5.2 mmol/l, BE: -12 mmol/l, Bicarb: 8 mmol/l. What specific therapies would you now use to treat this patient? Tick all that apply:**

- ☐ 2L IV fluid bolus
- ☐ Calcium replacement
- ☐ 20ml/Kg IV fluid bolus
- ☐ Sodium replacement
- ☐ Atropine boluses: up to 3 boluses in total
- ☐ Amiodarone infusion
- ☐ Adrenaline boluses: up to 4 boluses in total
- ☐ Inotropic infusion
- ☐ Intubation
- ☐ High dose insulin euglycaemic therapy
- ☐ Glucagon boluses and infusion

10. **The patient is now intubated and was started on an adrenaline infusion (0.5 µg/kg/hr) and HIET (1 IU/kg/hour). The nursing sister is concerned as patient’s vitals are: BP=88/53 mmHg, HR=48 BPM, SPO2= 94% on FiO2 of 60% and HGT is 12mmol/l. Which of the following is most likely to improve the patient’s clinical condition?**

- Increase FiO2 on the ventilator
- Replace the patient’s glucose
- Increase the HIET to 5IU/Kg/hour and monitor blood pressure changes
- Give 60mg of Lasix IV stat
- Consider haemodialysis for the patient

11. **The patient remains hypotensive despite the adrenaline infusion and HIET, which is currently running at 10 IU/kg/hr. What other therapies could you consider administering to this patient? Tick all that apply:**
    - ☐ Phosphodiesterase inhibitors
    - ☐ Amiodarone loading dose then infusion
    - ☐ Intralipid infusion
    - ☐ Cardiac pacing
    - ☐ Atropine infusion
    - ☐ Extracorporeal membrane oxygenation: ECMO
    - ☐ Albumin Haemodialysis
    - ☐ Other: please state

12. **Does glucagon play a role in treating calcium channel blocker overdose?**
    - o There is no role
    - o There is a role, but shouldn't be first choice
    - o There is a role as it improves mean arterial pressure
    - o I don't know

13. **What is the maximum recommended amount of intravenous fluid that should be given to a patient with symptomatic calcium channel blocker overdose?**

    - o 10 ml/kg
    - o 20 ml/kg
    - o 30 ml/kg
    - o 40 ml/kg
    - o 50 ml/kg
    - o 60 ml/kg

14. **What guidelines have been published to assist emergency doctors with managing a patient with a CCB overdose?**

    Open ended question

15. **Explain what you understand by the term high-dose insulin euglycaemic therapy (HIET) in the management of calcium channel blocker overdose?**

    Open-ended question: must contain
    - **High-dose insulin**
    - **Euglycemia**
    - **Used in CCB toxicity**
    - **Improve myocardial contractility**

16. **Where did you first learn about HIET?**

- Medical school
- Working in the Emergency Department
- Colleagues
- Social media
- Textbook
- Journal article
- I have never heard about it before now
- Other: please state

17. A 32-year-old male patient presents to your ED 8 hours post ingestion of an unknown amount of Amlodipine. His vitals are: BP= 80/58mm Hg, HR= 40 BPM, SPO2= 94% on RA and a GCS of 15/15. A CVP line has been inserted and adrenaline infusion started at 0.1 µg/kg/hr. The patient has already received a 20 ml/kg fluid bolus and 30 ml of calcium gluconate. When would you consider starting HIET on this patient?

- I would not start HIET on this patient
- Simultaneously with the adrenaline infusion
- Only after patient is accepted to ICU: 24 hours post ingestion of Amlodipine
- Once the patient has reached the maximum dose of the adrenaline infusion and remains haemodynamically unstable
- Only if patient requires intubation
- Once the patient has required adrenaline 0.5 ug/kg/min and remains haemodynamically unstable
- I don't know

18. Do you think there is strong evidence for the use of HIET as an effective clinical treatment?

- Yes
- No
- I'm not sure

19. What are commonly reported side effects of HIET? Tick all that apply:

- Hypoglycaemia
- Hypocalcaemia
- Hypomagnesaemia
- Hypotension
- Lactic acidosis
- Hypernatraemia
- Hypokalaemia
- Hyponatraemia
- Pulmonary oedema
- Other: please state

20. What is the starting dosage used in HIET?

- 0.1 IU/kg/hr
- 0.05 IU/kg/hr
- 1 IU/kg/hr

- Other: please state:

21. What is the commonly accepted ceiling dose used in HIET?

- 1 IU/kg/hr
- 10 IU/kg/hr
- 16 IU/kg/hr
- 22 IU/kg/hr

22. What is the maximum inotropic infusion dose in a toxic calcium channel blocker overdose patient?

Open ended question: no ceiling dose

23. What are the therapeutic end-points of HIET? Tick all that apply:

- Systolic blood pressure > 90 mmHg
- Heart rate > 40 bpm
- Acidemia resolution
- Hyperglycaemia
- Urine output: 1-2 ml/kg/hr
- Improved mental status
- Persistent cardiac conduction abnormalities
- Other: please state

Practice

The following section relates to emergency doctor practices in their current ED. There are no right or wrong answers.

24. How do you currently manage a CCB OD in your Emergency Department?

- Start inotropic infusion
- Start inotropic infusion and HIET and then refer to ICU
- Start HIET
- Refer immediately to ICU
- Do nothing

25. What specific vasopressor or inotrope would you prefer to initiate in a CCB OD?

- Adrenaline
- Dopamine
- Phenylephrine
- Dobutamine
- Other: Please state

26. When managing a calcium channel blocker overdose, from where would you most likely seek advice?

- Internal medicine registrar
- Emergency physician consultant
- Poison centre
- A more senior doctor on the floor
- I would not seek advice
- Other: please state

27. How many patients with a calcium channel blocker overdose have you treated in the past 12 months?

Insert number

28. Have you used HIET before?

- Yes
- No

If yes, please describe any problems you encountered when using HIET.

29. Would any of the following resource limitations decrease the likelihood of your decision to start HIET? Tick all that apply:

- Unable to Call poison Centre
- Unavailable consultant for advice
- Lack of ICU beds
- CVP line confidence
- Busy emergency department
- Full resuscitation bed status
- Understaffing in the unit
- Unavailability of medications needed
- Unavailability of infusion pumps
- Other: please state:

30. Does your current work environment have the required resources and expertise to effectively implement HIET?

31. Does your past experience with treating calcium channel blocker overdoses affect your decision to initiate HIET? If so, how?
