# Supplementary material for: Exploring the knowledge and practice of calcium channel blocker overdose management among South African Emergency Medicine doctors
Source: Afr J Emerg Med. 2026 Apr 1;16(2):100970. doi: 10.1016/j.afjem.2026.100970 (PMC13087764; doi:10.1016/j.afjem.2026.100970)
Supplement: Supplementary file 3 [file mmc3.pdf]

Appendix C: Social Media Advert

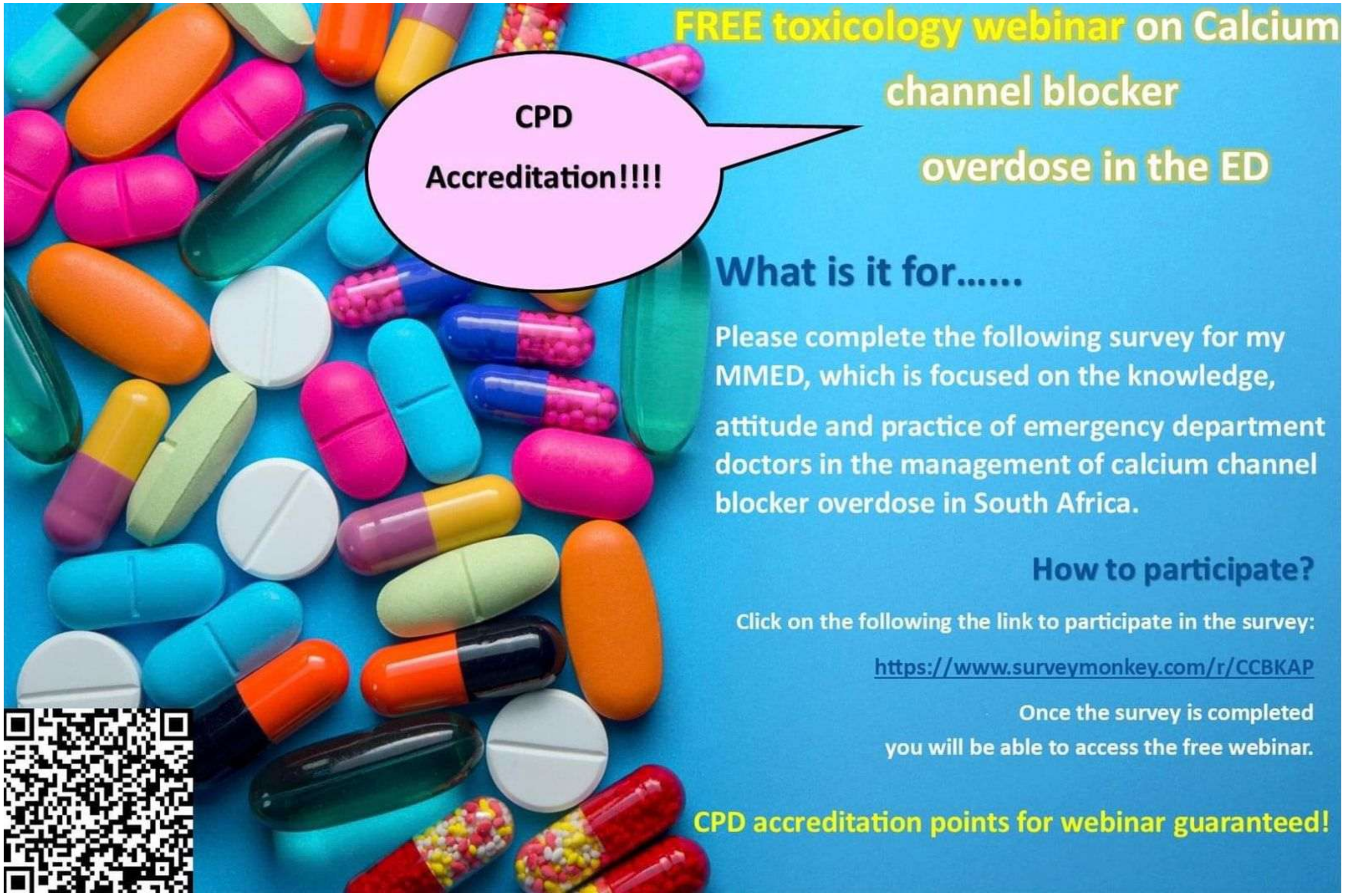

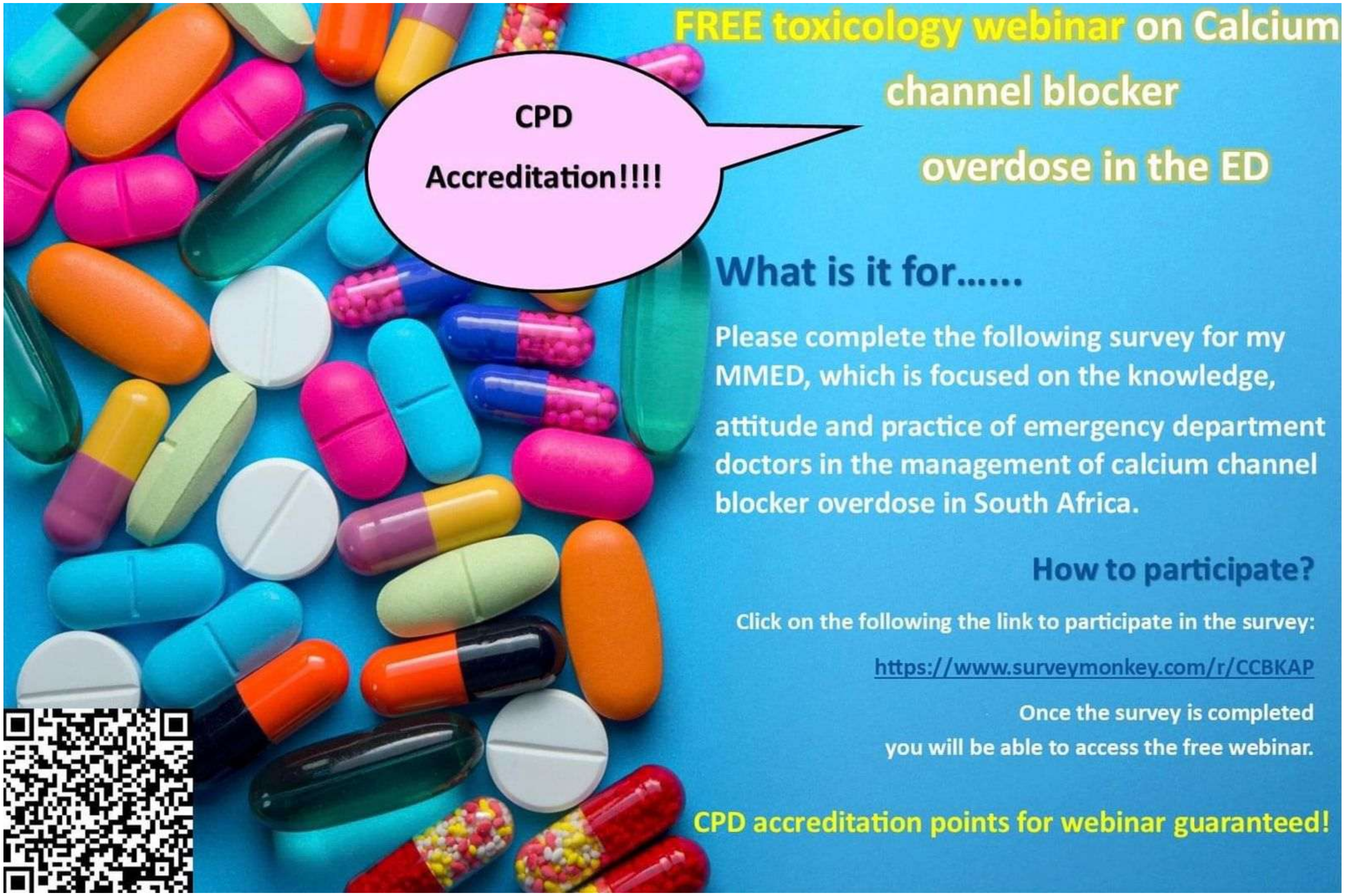
FREE toxicology webinar on Calcium
channel blocker
overdose in the ED
CPD
Accreditation!!!!
What is it for……
Please complete the following survey for my MMED, which is focused on the knowledge, attitude and practice of emergency department doctors in the management of calcium channel blocker overdose in South Africa.
How to participate?
Click on the following the link to participate in the survey:
https://www.surveymonkey.com/r/CCBKAP
Once the survey is completed
you will be able to access the free webinar.
CPD accreditation points for webinar guaranteed!
