# Supplementary material for: Exploring the knowledge and practice of calcium channel blocker overdose management among South African Emergency Medicine doctors
Source: Afr J Emerg Med. 2026 Apr 1;16(2):100970. doi: 10.1016/j.afjem.2026.100970 (PMC13087764; doi:10.1016/j.afjem.2026.100970)
Supplement: Supplementary file 4 [file mmc4.pdf]

Appendix D

Questionnaire Memo Score Sheet: Closed-ended Questions and Open-ended Questions

No	Answers						Marks (34)	Participants						
								1	2	3	4	5	6	7
6.	Hyperglycaemia	Hypotension	Depressed level of consciousness				(3)							
7.	20mg						(1)							
8.	Reliable IV access	Oxygenation using poly mask oxygen					(2)							
9.	Calcium Replacement	20ml/Kg IV fluid bolus	Atropine boluses: up to 3 in total	Inotropic infusion	Intubation	HIET	(6)							
10.	Increase the HIET to 5IU/Kg/hr and monitor blood pressure changes						(1)							
11.	Phosphodiestera se inhibitors	Intralipid infusion	Cardiac pacing	ECMO	Albumin haemodialysis		(5)							
12.	Glucagon has no role						(1)							
13.	20ml/Kg						(1)							
15.*	High dose insulin	euglycaemia	Used in CCB toxicity	To improve myocardial contractility			(2)							
17.	Simultaneously with the adrenaline infusion						(1)							
19.	Hypoglycaemia	Hypokalaemi a	Hyponatraemia	Hypomagnes ia			(4)							
20.	1 IU/Kg/hr						(1)							
21.	22 IU/Kg/hr						(1)							
22.*	No maximum inotropic dose because toxicology patient/ No ceiling dose						(1)							
23.	SBP >90 mmHg	Acidemia resolution	Urine output 1-2 ml/kg/hr	Improved mental status			(4)							

*Questions 15 and 22: Model answers (in red) represent the memo used to guide scoring. Responses were coded verbatim and assessed against these model answers for consistency in analysis.
